# Ultra-processed food advertisements dominate the food advertising landscape in two Stockholm areas with low vs high socioeconomic status. Is it time for regulatory action?

**DOI:** 10.1186/s12889-019-8090-5

**Published:** 2019-12-21

**Authors:** Petter Fagerberg, Billy Langlet, Aleksandra Oravsky, Johanna Sandborg, Marie Löf, Ioannis Ioakimidis

**Affiliations:** 1 Innovative Use of Mobile Phones to Promote Physical Activity and Nutrition Across the Lifespan (the IMPACT) Research Group, Department of Biosciences and Nutrition, Karolinska Institutet, 14152 Stockholm, Sweden; 2 Department of Medical and Health Sciences, Linköping University, Linköping, Sweden

**Keywords:** Outdoor advertising, Food advertising, Obesity, Ultra-processed food, Sugary drinks, Sugar, Sweetened beverages (SSB), Discretionary food, Fast food, Sustainability

## Abstract

**Background:**

Ultra-processed food consumption is a risk factor for obesity and has a negative environmental impact. Food companies spend billions of dollars on advertisements each year to increase the consumption of ultra-processed food. In Australia, USA, and New Zealand, most food advertisements around schools and in train stations promote ultra-processed food, but no similar studies have been conducted in Sweden. The aim of this study was to explore the proportion of ultra-processed food advertisements in two districts of Stockholm, Sweden with low vs. high socioeconomic status (SES).

**Methods:**

Two independent researchers (per area) mapped all advertisements, including storefronts, in two Stockholm districts. During consecutive days, all advertisements were photographed in Skärholmen (low SES district), and Östermalmstorg (high SES district), on the streets inside and outside the subway stations, as well as inside and outside of local shopping malls. Advertisements promoting food products were identified and a trained dietician categorized whether they promoted ultra-processed foods. Chi-Square test was conducted to test for differences in the proportion of ultra-processed food advertisements between the two study areas.

**Results:**

In total, 4092 advertisements were photographed in Skärholmen (*n* = 1935) and Östermalm (*n* = 2157). 32.8% of all advertisements promoted food, while 65.4% of food advertisements promoted ultra-processed foods. A significantly higher proportion of ultra-processed food advertisements out of total food advertisements was identified in the low SES area, irrespective of the researcher taking the pictures (74.6% vs. 61.8%, *p* < 0.001 and 70.4% vs. 54.8%, *p* = 0.001). There was no significant difference in the proportion of food advertisements out of total advertisements between the two areas.

**Conclusions:**

This study provides initial evidence about the scale and the differences in exposure to food advertisements across areas in Stockholm. The observed high proportion of ultra-processed food advertisements is concerning and is in sharp contrast to the Swedish dietary guidelines that recommend reduced consumption of such foods. Based on our results, residents in low SES areas might be more exposed to ultra-processed food advertisements than those in high SES areas in Stockholm. If such findings are confirmed in additional areas, they should be considered during the deployment of food advertisement regulatory actions.

## Introduction

Today, obesity is one of the greatest public health threats in the world. In fact, obesity is associated with early death [1], type 2 diabetes [2], coronary artery disease [2], cancer [3] and depression [4], while the global economic burden of obesity has been estimated to be equivalent to that of smoking or armed violence, war, and terrorism added together [5]. Furthermore, the public health threat of obesity is growing, as its global prevalence has been steadily increasing from 3.2 and 6.4% in 1975, among men and women respectively, to 10.8 and 14.9% in 2014 [6]. This observation indicates that the problem might further worsen in the coming decades, if the trend continues.

The main cause of obesity is overconsumption of energy from foods and drinks relative to energy needs [7, 8]. A food type that has been shown to promote overconsumption of energy and subsequent weight gain is ultra-processed foods (i.e. fast foods such as sugary drinks, junk foods and convenience foods). Indeed, recent epidemiological studies have linked ultra-processed foods to both weight gain and increased obesity prevalence [9]. In addition, the affordability of sugary drinks has been suggested to be the major driver of increased sugary drink purchase and consumption, which in turn is associated with higher levels of obesity prevalence [10]. Interestingly, the results of a recent randomized crossover trial, in a tightly controlled metabolic ward setting, strongly support that this relationship is causal, i.e. consumption of ultra-processed foods results in higher energy intake, subsequently leading to increases in body weight [11].

Furthermore, changes in the global food system have been associated with the ongoing climate crisis [12], with ultra-processed foods being identified as one of the most important diet-related environmental footprints [13]. Additionally, commercial actors, i.e. producers of ultra-processed foods, market their products aggressively and interfere with implementation of policies and regulations for the improvement of public and planetary health [12, 14].

In order to regulate the promotion of food and beverages, the International Chamber of Commerce (ICC) has provided a responsible communications framework, stating that “*marketing communications should not undermine the importance of healthy lifestyles*” and “*claims should be conveyed consistent with the nature and scope of the evidence, providing the consumer with supportable information*” [15]. Furthermore, in the advertising and marketing communications code [16], ICC states that “*Information provided with the product should include proper directions for use and full instructions covering health and safety aspects whenever necessary. Such health and safety warnings should be made clear by the use of pictures, sound, text or a combination of these*”. However, currently, no such information exists on ultra-processed food packaging (i.e. that consumption increases the risk for obesity and related diseases), even though ultra-processed food producers claim that they adhere to this framework, i.e. [17].

In Sweden, approximately half of the population is now overweight or obese, following the global trend, and localized obesity prevalence differences are not uncommon [18]. In the capital itself, Stockholm, similar regional/neighborhood differences in obesity prevalence have been observed [19, 20]. Among 4-year-old children (born 2013), the prevalence of overweight and obesity ranges from 6.5–17.4% in different areas of Stockholm [19]. For example, in Skärholmen, an area with a high percentage of children belonging to households with non-Western background and low buying power, 14.3% of 4-year-old children born 2013 were overweight or obese 2017. On the other hand, Östermalm, an area with a high percentage of children belonging to households with Swedish background and high buying power, only 7.3% of the 4-year-old children were overweight or obese 2017. In fact, the buying power of children’s households has a strong negative correlation with obesity rates across the Stockholm region [19]. Similarly, among adults aged 18–64 years, only 5.9% were obese in Östermalm 2015 vs. 18.2% in Skärholmen [20].

A potential cause of the observed regional differences (globally and in Sweden) might be different rates of exposure and availability of food and beverage advertisements. However, across the literature, most studies have been conducted on television advertising, with only a limited amount of studies done on outdoor advertisements. Taken together [21–29], the literature on outdoor food advertisements suggests that most food ads promote “unhealthy” foods, such as sugary beverages and fast foods (i.e. ultra-processed foods). Additionally, disadvantaged groups of residents seem to be targeted more heavily than others, potentially affecting their food purchasing habits.

Unfortunately, no studies on outdoor food advertisements have been conducted in Sweden and only one study has been conducted in the European context (Newcastle UK) [29]. Furthermore, no studies have examined potential differences in outdoor food advertisement exposure in areas of low and high socioeconomic status in Sweden.

Therefore, the aim of this study was to explore the proportion of advertisements directly related to ultra-processed foods (including sugary drinks and fast foods) in two areas of Stockholm, Sweden; 1) Skärholmen, an area with relatively high obesity prevalence and low socio-economic status, and 2) Östermalm, an area with relatively low obesity prevalence and high socio-economic status. This study is an important addition to the food advertising literature since risk factors for diseases have been shown to differ both between and within countries [8, 30] and no comparable study has been conducted in Sweden.

## Methods

### Study design

A cross-sectional study design was used to examine the advertisements in the two included areas.

### Setting

**Fig. 1:**
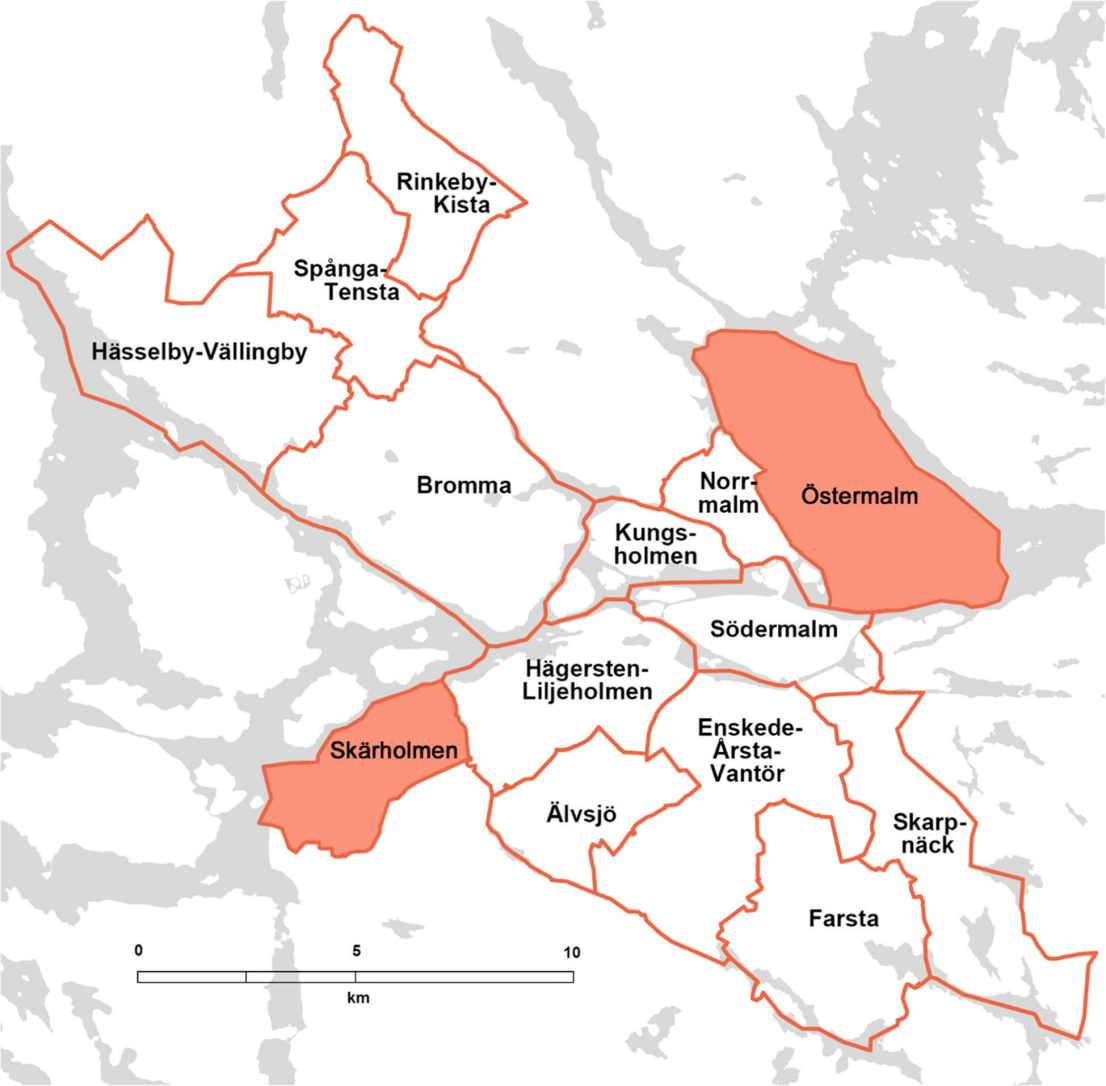
Map showing Stockholm municipality and its districts. The selected analyzed areas are colored orange (Östermalm and Skärholmen). Adopted and modified (with permission) from [31]

The study took place in Skärholmen and Östermalm, two districts located in Stockholm municipality (see Fig. 1) that is part of Stockholm county, Sweden. These areas represent low and high socio-economic status areas as well as areas with disadvantaged vs. advantaged residents, respectively.

More specifically, a higher proportion of residents in Skärholmen are foreign born or born in Sweden with both parents born abroad (70% vs. 22%), unemployed (6% vs. 1.5%), uses sickness cash benefit and disability pension (26% vs. 10%) compared to Östermalm. At the same time, a lower proportion of residents in Skärholmen vs. Östermalm have tertiary education (38% vs. 72%) and the mean income is approximately half of the mean income in Östermalm (256.800 SEK/year vs. 505.500 SEK/year) [19, 31].

### Inclusion criteria

The two areas were selected based on the local reported overweight and obesity prevalence [19, 31] and the fact that both areas have a subway station that is in close proximity to a shopping mall (within 100 m distance), thus making them reasonably comparable in advertisement exposure potential in these settings [32, 33].

### Data collection procedure

On Wednesday 30th of May 2018 (Day 1, starting at ~ 11.00 CET), two independent researchers (researcher 1 and researcher 2) travelled to Skärholmen, and on the next day, 1st of June 2018 (Day 2, also starting ~ 11.00 CET), researcher 1 and another independent researcher (researcher 3) travelled to Östermalmstorg to conduct the advertisement documentation process.

On day 1, all advertisements (including storefronts) in the subway station Skärholmen (including all advertisements 50 m to the left and right on the streets outside each of the entrances to the subway station) were documented by both researcher 1 and 2. Thereafter, all advertisements inside the shopping mall “SKHLM” (located within 100 m proximity to Skärholmen subway station) were documented as well as all advertisements on the streets surrounding the shopping mall.

On day 2, the same procedure was repeated by researcher 1 and 3 in the subway station Östermalmstorg, the streets outside of the entrances to the subway station as well as inside and outside the shopping mall “Sturegallerian” (located within 100 m proximity to Östermalmstorg subway station).

We defined an advertisement as something that is shown or presented to the public to help sell a product or to make an announcement. If several advertisements were placed in a store window for promotion purposes, one picture including all products/advertisements seen in each store window was taken. If several advertisements were included on one poster (i.e. advertisements for discounted food products outside of supermarkets), then one picture per poster was taken. For rotating billboards, all advertisements shown within one complete rotation were recorded. No pictures of flyers or brochures were taken, neither were advertisements inside/outside moving vehicles (i.e. busses and subway trains). Similarly, advertisements placed inside stores were not recorded.

### Data collection equipment

To enable documentation of advertisements, smartphones with built-in cameras were used. Researcher 1 used Apple iPhone 7, researcher 2 LG G3 and researcher 3 used LG G6. All pictures of advertisements were later exported to a personal computer and sorted into folders for each researcher and location.

### Advertisement categorization process

**Fig. 2:**
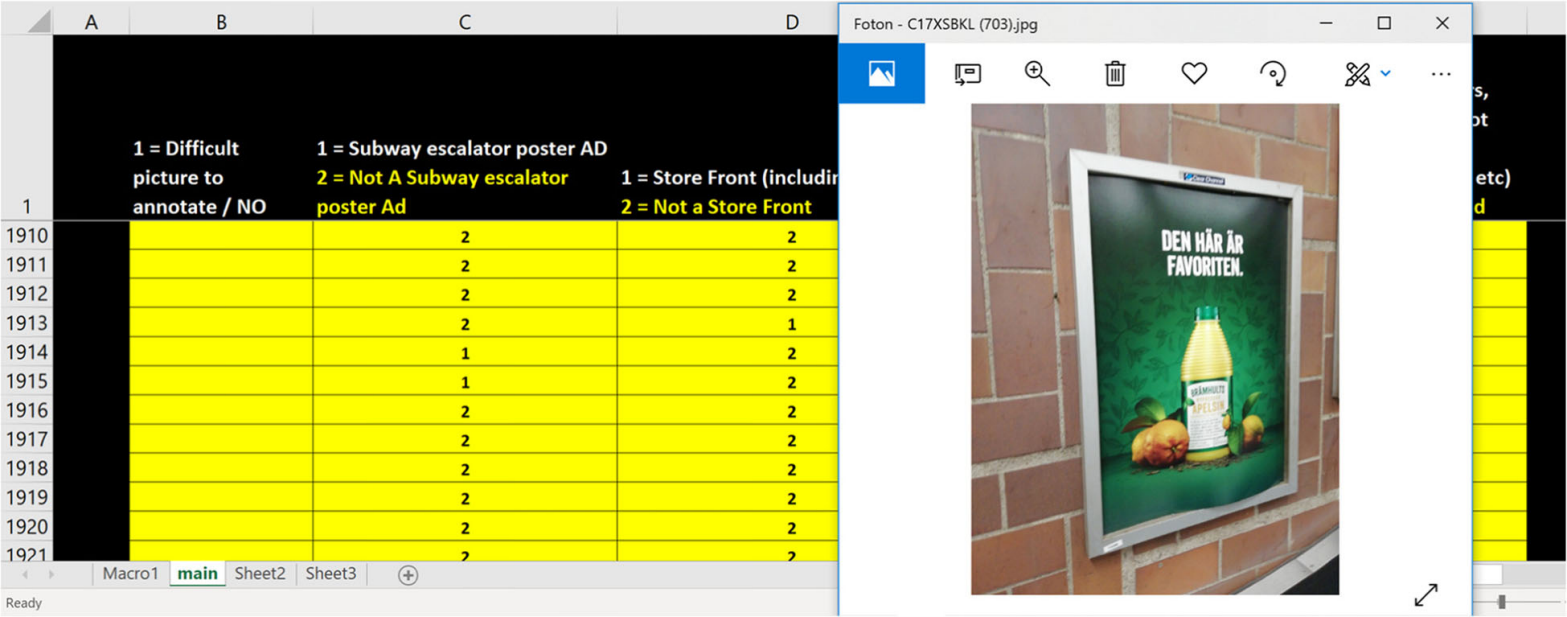
Screenshot of the Excel file and how each picture went through the annotation process

To facilitate the categorization process, an in-house built Excel (Microsoft®) tool was used (see Fig. 2), randomly ordering picture links in separate Excel rows. Thus, all the collected pictures (taken by any researcher, during any of the two days) were accessed in random order. Each picture was marked by a hidden label (see black cells in column A in Fig. 2) encoding the identity of the researcher taking the picture and the originating area (Östermalm or Skärholmen). This enabled blinding of the “categorizer” (i.e., an independent, trained dietitian who did not participate in the data collection) about the location of each picture and the identity of the researcher who took it during the categorization process.

**Fig. 3:**
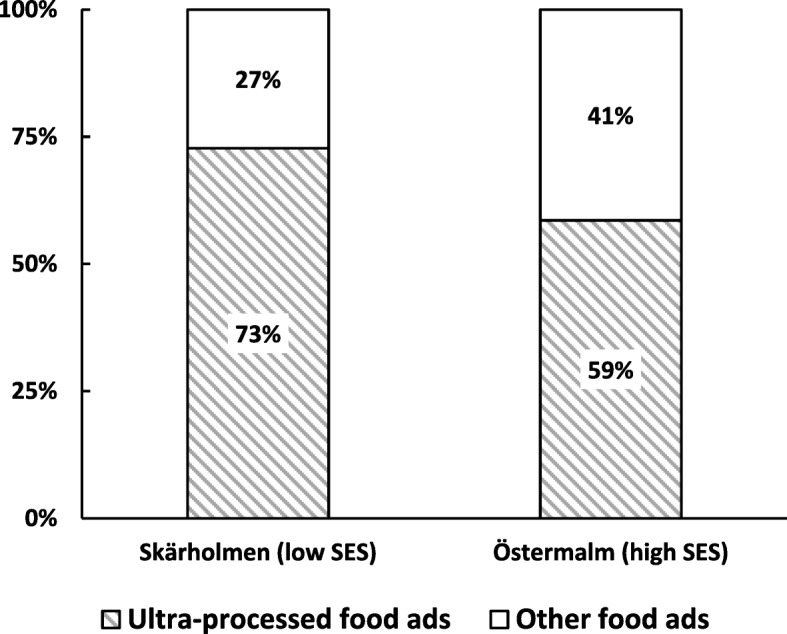
Proportion of ultra-processed food advertisement out of total food advertisements in the two chosen areas of Stockholm (Skärholmen and Östermalm). Advertisements in subway escalators are excluded. SES = socio-economic status, ads = advertisements

**Fig. 4:**
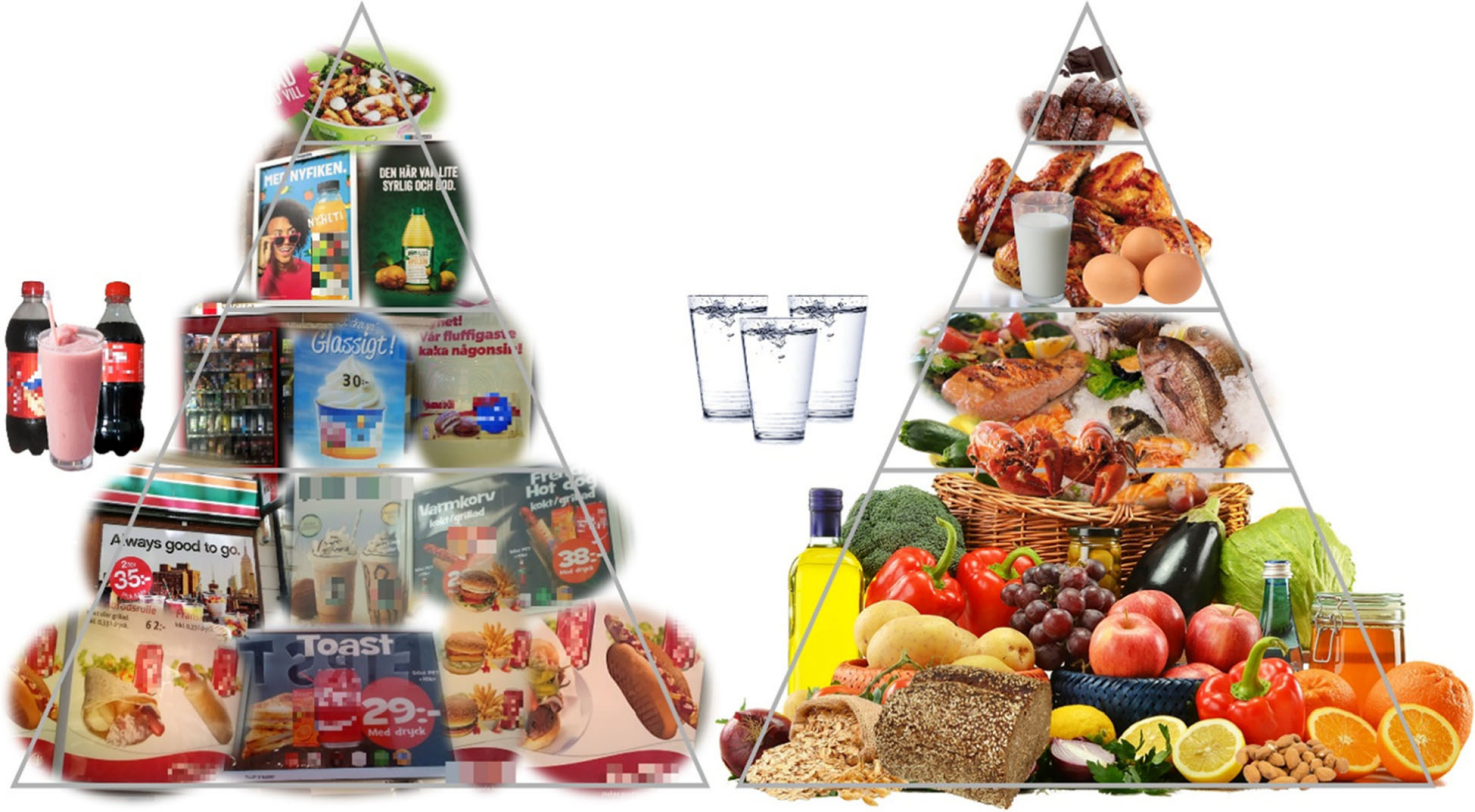
A stylized illustration of recommendations for a healthy diet (food pyramid to the right) vs. a sample of food advertisements in the included districts in Stockholm municipality (food pyramid to the left). The pyramid style was inspired by [45]

By clicking on a link in the Excel-file, the picture was loaded in the Microsoft Windows program “Photos” (see Fig. 2). Thereafter, the categorizer annotated each picture based on the following characteristics: 1) difficulty to annotate (see column B in Fig. 2), 2) being a subway escalator advertisement or not (see column C in Fig. 2), 3) depicting a store front or not (see column D in Fig. 2), 4) being an advertisement promoting food or not (see column E in Fig. 2), 5) including a sugary drink or not, and lastly, 6) including fast food promotion or not (see column G in Fig. 2). The fast food label was given to typical Swedish fast food items, such as hamburgers, kebab, pizza, hot dog, ice cream, pastries, candy and similar items. Based on this categorization process, for example, a picture of an advertisement promoting a fast food item together with a sugary drink would receive both labels (i.e., “fast food” and “sugary drink”), thus contributing to both categories. A single trained dietician who didn’t participate in the data collection categorized all the collected pictures. Finally, after the picture categorization process had been performed, another binary category was created (ultra-processed food) by adding the sum of “sugary drink” + “fast food” categories, in accordance with the Group 4 of the NOVA classification system [34], which includes soft drinks, sweet or savoury packaged snacks and reconstituted meat products (among others).

The categorization of subway escalator advertisements was added due to the special periodical, non-permanent nature of escalator advertisements in Stockholm (these change on weekly basis as stated by the advertisement company handling Stockholm subway advertising [35]). Indeed, the inclusion of such advertisements has the potential of significantly skewing the final analysis, due to the varied thematic of different advertising campaigns. Thus, the subway escalator advertisements were excluded from the final analysis. For example, during the data collection week, there was an ongoing advertising campaign specifically targeting the subway escalators in Östermalm. This campaign promoted a specific sugar sweetened juice (MER®, owned by The Coca-Cola Company [36]) at > 100 advertisement spots in Östermalm subway escalators. The results of the performed analysis including subway escalator advertisements can be found in “Additional file 1”.

### Reliability of the categorization process

To assess the reliability of ultra-processed food categorization, a random subset of the pictures belonging to the total picture dataset, consisting of 1000 pictures, was created and analyzed repeatedly by different categorizers. Thus, this sample was analyzed independently by the trained dietician and another trained researcher, to allow calculation of categorizer agreement (reliability).

### Statistical analysis

Chi-Square test was conducted to test differences in ultra-processed food proportions between the two study areas. *P* < 0.05 was considered as the threshold for significance.

Cohen’s Kappa (K) was used to evaluate inter-rater agreement. Kappa (K) value of 0–0.20 was interpreted as “No agreement”, 0.21–0.39 “Minimal agreement”, 0.40–0.59 “Weak agreement”, 0.60–0.79 “Moderate agreement”, 0.80–0.90 “Strong agreement”, and above 0.90 as “Almost perfect agreement” [37].

SPSS 25 (IBM, Armonk, NY, USA) software [38] was used to test differences in proportions of advertisements, while descriptive statistics were calculated in Microsoft® Excel.

## Results

### Descriptive statistics

In total, 4092 pictures of advertisements (ads) originating from Skärholmen (*n* = 1935) and Östermalm (*n* = 2157) were included in the final analysis. This dataset excludes subway escalator ads (*n* = 443) and pictures that were impossible to annotate (i.e. very blurry pictures, *n* = 8). Out of the ads included in the final dataset, 1341 pictures (32.8% of total ads) were ads promoting food products (33.1% in Skärholmen vs. 32.5% in Östermalm), while 877 pictures (65.4% of all food ads) were of ads promoting ultra-processed food products (i.e. sugary drinks, hamburgers, hot dogs, kebab, candy, ice cream etc.). More specifically, in Skärholmen, 466 pictures (73% of total food ads) were ads promoting ultra-processed foods vs. 411 (59% of total food ads) in Östermalm (See Fig. 3).

### Test of ad proportions

There was no significant difference between Skärholmen and Östermalm in proportion of food ads out of total ads (Researcher 1: 34.9% vs. 35.1%, *p* = 0.924, χ^2^ = 0.009; Researcher 2 + 3: 31.1% vs. 29.9%, *p* = 0.555, χ^2^ = 0.348 respectively). However, Skärholmen had a significantly higher proportion of ads promoting ultra-processed food out of total food ads (Researcher 1: 74.6% vs. 61.8%, *p* < 0.001, χ^2^ = 13.869; Researcher 2 + 3: 70.4% vs. 54.8%, *p* = 0.001, χ^2^ = 15.675). More detailed data of the ads photographed in both areas are presented in Table 1.

**Table 1:** Overview of the advertisements recorded at the two locations

		Östermalm	Skärholmen	Both areas
R1	Total ads	1072	1017	2089
	Food ads (% of total ads)	377 (35%)	355 (35%)	732 (35%)
	Ultra-processed food ads (% of food ads)	233 (62%)	265 (75%)	498 (68%)
	Fast food ads (% of ultra-processed food ads)	184 (79%)	232 (88%)	416 (84%)
	Sugary drink ads (% of ultra-processed food ads)	106 (45%)	109 (41%)	215 (43%)
R2 + R3	Total ads	1085	918	2003
	Food ads (% of total ads)	324 (30%)	285 (31%)	609 (30%)
	Ultra-processed food ads (% of food ads)	178 (55%)	201 (70%)	379 (62%)
	Fast food ads (% of ultra-processed food ads)	118 (66%)	174 (87%)	292 (77%)
	Sugary drink ads (% of ultra-processed food ads)	99 (56%)	83 (41%)	182 (48%)

R1 = pictures taken by Researcher 1, R2 + R3 = pictures taken by Researcher 2 and 3. Researcher 1 and Researcher 2 took pictures of advertisements on the same day in Skärholmen and Researcher 1 and Researcher 3 took pictures of advertisements in Östermalm on the next day

The observed differences in the proportion of ultra-processed food ads between the two areas were still present when including subway escalator ads in both analyses [see Additional file 1]. The observed difference in ultra-processed food ads could be explained by a higher proportion of fast food ads out of total food ads in Skärholmen (Researcher 1: 65.4% vs. 48.8%, *p* = 0.000, χ^2^ = 20.402; Researcher 2 + 3: 61.1% vs. 36.4%, *p* < 0.001, χ^2^ = 36.863), but not by differing proportions of sugary drink ads out of total food ads (Researcher 1: 30.7% vs. 28.1%, *p* = 0.442, χ^2^ = 0.590; Researcher 2 + 3: 29.1% vs. 30.6%, *p* = 0.700, χ^2^ = 0.149).

### Reliability of advertisement categorization

There was “almost perfect agreement” between the two raters for the categorization of subway escalator advertisements out of total ads (κ = 0.958). “Strong agreement” was observed for the categorization of food advertisements out of total ads (κ = 0.899) and sugary drinks out of total food ads (κ = 0.803). Finally, there was “moderate agreement” for the categorization of ultra-processed food ads out of total food ads (κ = 0.695) and for fast food ads out of total food ads (κ = 0.772).

## Discussion

This is the first study to examine outdoor food and ultra-processed food advertisements in Stockholm, Sweden and is the first attempt to investigate potential differences between two districts with low vs. high socioeconomic status in the municipality of Stockholm. The results suggest that a high proportion (~ 60–70%) of food advertisements, in these areas, are promoting ultra-processed foods and that disadvantaged districts may be more exposed to such advertisements than advantaged districts.

The high prevalence of ultra-processed food advertisements among the total food advertisements is in line with observations made in train stations on the Sydney metropolitan train network [25], subway lines in Bronx, US [22], around primary schools in Wollongong and Sydney, Australia [26], around schools in urban and rural regions in New Zealand [27] and around public and private elementary schools in Cuernavaca and Guad alajara, Mexico [28]. This should not come as a surprise, taking into consideration the total advertising budgets of major food companies. For example, the two biggest global companies in the market segment of sugary drinks, dedicated > 8 billion US dollars on product promotion in 2018 alone [39, 40].

However, these observations raise public health concerns since ultra-processed foods have been shown to cause overconsumption of energy and weight gain in humans [11], thus increasing the long-term risk of obesity and related negative consequences [1–5]. These concerns are even identified and discussed in the annual reports of major food companies [39, 40]. Additionally, epidemiological studies suggest a link between ultra-processed foods and increased risk of cardiovascular, coronary heart and cerebrovascular diseases [41], cancer [42], and all-cause mortality independently of BMI [43], as well as contributing to a negative environmental footprint [12, 13].

Furthermore, the overwhelming proportion of ultra-processed food ads observed in our study are incompatible with the national nutritional guidelines in Sweden (Nordic Nutrition Recommendations [44]), which recommend the reduction of ultra-processed foods intake, promoting increased intake of fruits, vegetables, berries, nuts, seeds, whole grains, fish and vegetable oils instead (see Fig. 4) [44].

This great disconnect between Swedish nutritional recommendations and actual food advertisements in the two analyzed areas is quite striking. These results, mirrored by the observations in other countries, indicate that the “self-regulatory system” of the food industry has probably been ineffective in their “attempt” to adhere to the framework for responsible food and beverage marketing communications, as described by the International Chamber of Commerce (ICC) [15]. As noted before [46], additional, externally enforced regulations of food and beverage advertisements might be needed in order to ensure the adherence of food companies to responsible food marketing. From the industry side, the global introduction of such regulations has been described as a potential risk to product sales and long-term profitability (e.g., [39, 40]), while the involved companies have previously tried to undermine the role of sugary drink consumption on national and global obesity prevalence [14]. Similarly, the food industry has also noted that additional labeling, warning requirements and/or increased taxes aimed at specific food products might reduce sales [39, 40]. However, regulatory actions to reduce tobacco use, at least in Sweden, correlate with a reduction of the percentage of smokers from 27 to 11% (1989 vs. 2017 respectively) [47–49] and, currently, a similar public discussion for the regulation of gambling advertisements is ongoing [50]. Therefore, similar actions could potentially be useful to reduce ultra-processed food consumption and long-term obesity rates as well as reducing the climate impact from the production and distribution of these types of food products [12, 13].

Additionally, the observation of a higher proportion of ultra-processed food advertisement in the low vs. the high socioeconomic areas in Stockholm, points to an additional public health concern. In these areas, childhood obesity prevalence is several times larger (4% vs. 0.4%) in the district with low vs high socioeconomic status [19], and three times higher (18% vs. 6%) among adults [20]. At the same time, national data on both adolescents and adults have shown that more disadvantaged groups in Sweden have lower nutritional quality vs. more advantaged groups [51, 52], with higher percentages of unhealthy food advertising in disadvantaged areas potentially contributing to the issue. It is important to add that a connection between socioeconomic status in an area (and/or living in neighborhoods with disadvantaged groups) and advertising of unhealthy food products have been observed before (i.e. in Australia [25], Bronx, New York, US [22], Los Angeles, Austin, New York and Philadelphia US [23], central Texas, US [24], Cuernavaca and Guadalajara, Mexico [28], and Wellington and Wairarapa New Zealand [27]), pointing toward the global character of the issue. Thus, it might be useful for novel food advertising regulations to acknowledge the socioeconomic and geographical aspects of the problem when they are deployed. In Sweden, in the domain of smoking, all the segments of the population (in regard to their educational level) seem to have been positively affected by the public anti-smoking regulations [53].

In the current study, the performed analysis of reliability showed “moderate agreement” for categorizing the variable ultra-processed food ads out of total food ads, highlighting the need for rigorous standards when categorizing food ads. In our case, we intentionally created a food advertisement protocol that removed the need of categorizing food advertisements on the recording spot (as previously done by others [26]). That allowed us to introduce another measure of unbiased advertisement categorization by “blinding” the trained dietician regarding the original location of each advertisement. Additionally, the selected protocol, based on recorded advertisement pictures, also facilitated better quality control of the categorizing process by independent researchers. For example, pictures categorized as ultra-processed food advertisements could be accessed by an independent researcher for re-evaluation. This process might be even more important in less diverse areas, i.e. when the difference in proportions of ultra-processed food is smaller between areas that are being compared. Additionally, our process allows for a more detailed analysis of the advertisements in the future (in accordance to previously suggested protocols in the Nordic countries [54]), i.e. restaurant ads, supermarket ads and specific brand logos etc. Finally, picture-based recording of advertisements may also accommodate an increased number of less trained researchers to record pictures, while the categorization of the advertisement is performed afterwards by dedicated research personnel. Thus, such a protocol is more scalable and could be considered in a “citizen science” data collection framework. Indeed, efforts for the collection of Big Data on behavioral and living environment parameters and their association with obesity prevalence are already ongoing [55]. The current study protocol, with minimal adaptations, could be a natural fit to such endeavors, providing data for use by public health authorities, as well as for the creation of educational opportunities through the organization of citizen science projects in collaboration with schools [56].

The subway escalator ad campaign observed in our study shows that the proportion of ultra-processed food ads can vary from one week to another in areas with subway stations, at least in Stockholm. In our study, this did not change the obtained results (i.e. proportion of ultra-processed food advertisements out of total food ads were still significantly higher in the district with low socioeconomic status vs. the one with high socioeconomic status [see Additional file 1]), but might be important if districts with more similar socioeconomic status are compared. In such case, repeated measures of ads would be needed to get a more representative view of the actual ultra-processed food advertisement exposure in the selected areas.

Our study has some limitations. To begin with, we conducted a cross-sectional analysis of food advertisements. Our results are therefore limited to the point in time of our data collection. Furthermore, we only included two out of fourteen available districts in Stockholm municipality. These districts were selected to represent socioeconomic extremes (i.e. relatively low average income vs. high, relatively low educational level vs. high etc.). It would be interesting to include more districts in Stockholm to evaluate if there are similar trends in ultra-processed food advertising in other areas as well. Adding to this point, we chose to analyze ads near the main subway station in each of the included districts, as well as the main local shopping mall. Future studies could therefore expand data collection to all subway stations and malls in an area to get a more complete picture of ad exposure. Additionally, ads were mapped during one week in the summer only, while a past report [25] have identified significant seasonal changes, with increased “unhealthy” food advertising during summer months, agreeing with food company reports that point to a seasonal variation of ultra-processed product sales [39, 40]. Finally, similarly to the majority of this field, our study focused on a static quantification of food advertisement presence in certain geographical areas, without quantifying their exposure effects (elsewhere referred to as “viewership” [57]). In the future, use of eye-tracking technology in the proposed protocol could further increase the analytical power. More concretely, level of attention on ultra-processed food advertisements vs. other food ads could be investigated.

## Conclusions

Our study reveals that two districts of low vs high socioeconomic status in Stockholm municipality are being exposed to a high proportion of ultra-processed food advertisements out of total food ads. This observation is in sharp contrast to national dietary guidelines and regulatory action is suggested to reverse the current exposure to ultra-processed food ads. Additionally, our results indicate that residents in low socioeconomic status areas might be more exposed to ultra-processed food advertisements than residents in areas with high socioeconomic status in Stockholm. If such findings are confirmed in additional areas in Stockholm and across Sweden, they should be taken in consideration upon deployment of novel public health measures on food advertising. In the future, further optimizations in the data collection protocols might facilitate larger scale studies, describing the local food advertisement landscape across wider geographical areas.

## Supplementary information

**Additional file 1.** Proportion of ultra-processed food ads out of total food ads between the two areas when including subway escalator ads.

## Data Availability

Data are available on a reasonable request.

## Abbreviations

Ads: Advertisements
SES: Socioeconomic status
