# Supplementary material for: Ultra-processed food advertisements dominate the food advertising landscape in two Stockholm areas with low vs high socioeconomic status. Is it time for regulatory action?
Source: BMC Public Health. 2019 Dec 21;19:1717. doi: 10.1186/s12889-019-8090-5 (PMC6925898; doi:10.1186/s12889-019-8090-5)
Supplement: Supplementary file 1 — Additional file 1. Proportion of ultra-processed food ads out of total food ads between the two areas when including subway escalator ads. [file 12889_2019_8090_MOESM1_ESM.docx]

Supplementary material

# Researcher 1. Skärholmen vs. Östermalm % ultra Processed food ads out of total FOOD ads.

# INCLUDES SUBWAY ESCALATOR PICTURES

| **Ultra processed food (1) * Area (1) Crosstabulation** | | | | | |
| --- | --- | --- | --- | --- | --- |
|  | | | Area (1) | | Total |
|  |  |  | Östermalm | Skärholmen |  |
| Fast food/sugary drink (1) | Not Ultra Processed food | Count | 154 | 93 | 247 |
|  |  | % within Area (1) | 34.0% | 25.0% | 29.9% |
|  | Ultra processed food | Count | 299 | 279 | 578 |
|  |  | % within Area (1) | 66.0% | 75.0% | 70.1% |
| Total | | Count | 453 | 372 | 825 |
|  |  | % within Area (1) | 100.0% | 100.0% | 100.0% |

| **Chi-Square Tests** | | | | | |
| --- | --- | --- | --- | --- | --- |
|  | Value | df | Asymptotic Significance (2-sided) | Exact Sig. (2-sided) | Exact Sig. (1-sided) |
| Pearson Chi-Square | 7.880^a^ | 1 | .005 |  |  |
| Continuity Correction^b^ | 7.457 | 1 | .006 |  |  |
| Likelihood Ratio | 7.948 | 1 | .005 |  |  |
| Fisher's Exact Test |  |  |  | .006 | .003 |
| Linear-by-Linear Association | 7.871 | 1 | .005 |  |  |
| N of Valid Cases | 825 |  |  |  |  |
| a. 0 cells (0.0%) have expected count less than 5. The minimum expected count is 111.37. | | | | | |
| b. Computed only for a 2x2 table | | | | | |

# Researcher 2. Skärholmen vs. Östermalm % ultra Processed food ads out of total FOOD ads.

# INCLUDES SUBWAY ESCALATOR PICTURES

| **Ultra processed food (1) * Area (1) Crosstabulation** | | | | | |
| --- | --- | --- | --- | --- | --- |
|  | | | Area (1) | | Total |
|  |  |  | Östermalm | Skärholmen |  |
| Fast food/sugary drink (1) | Not Ultra Processed food | Count | 155 | 86 | 241 |
|  |  | % within Area (1) | 38.6% | 28.9% | 34.4% |
|  | Ultra processed food | Count | 247 | 212 | 459 |
|  |  | % within Area (1) | 61.4% | 71.1% | 65.6% |
| Total | | Count | 402 | 298 | 700 |
|  |  | % within Area (1) | 100.0% | 100.0% | 100.0% |

| **Chi-Square Tests** | | | | | |
| --- | --- | --- | --- | --- | --- |
|  | Value | df | Asymptotic Significance (2-sided) | Exact Sig. (2-sided) | Exact Sig. (1-sided) |
| Pearson Chi-Square | 7.130^a^ | 1 | .008 |  |  |
| Continuity Correction^b^ | 6.707 | 1 | .010 |  |  |
| Likelihood Ratio | 7.197 | 1 | .007 |  |  |
| Fisher's Exact Test |  |  |  | .008 | .005 |
| Linear-by-Linear Association | 7.120 | 1 | .008 |  |  |
| N of Valid Cases | 700 |  |  |  |  |
| a. 0 cells (0.0%) have expected count less than 5. The minimum expected count is 102.60. | | | | | |
| b. Computed only for a 2x2 table | | | | | |
